# Imputing not available values in single‐cell DNA methylation data using the median is straightforward and effective

**DOI:** 10.1002/qub2.70000

**Published:** 2025-04-01

**Authors:** Songming Tang, Siyu Li, Shengquan Chen

**Affiliations:** ^1^ School of Mathematical Sciences and LPMC Nankai University Tianjin China

**Keywords:** data imputation, single‐cell DNA methylation

## Abstract

Recent advances in single‐cell DNA methylation have provided unprecedented opportunities to explore cellular epigenetic differences with maximal resolution. A common workflow for single‐cell DNA methylation analysis is binning the genome into multiple regions and computing the average methylation level within each region. In this process, imputing not available (NA) values which are caused by the limited number of captured methylation sites is a necessary preprocessing step for downstream analyses. Existing studies have employed several simple imputation methods (such as zeros imputation or means imputation), however, there is a lack of theoretical studies or benchmark tests of these approaches. Through both experiments and theoretical analysis, we found that using the medians to impute NA values can effectively and simply reflect the methylation state of the NA values, providing an accurate foundation for downstream analyses.

DNA methylation (DNAm) is one of the earliest identified types of epigenetic modification and plays an essential role in regulating normal cellular processes, embryogenesis, and tumor development and progression [1, 2]. Recent advances in single‐cell DNA methylation (scDNAm) have provided unprecedented opportunities to explore cellular epigenetic differences with maximal resolution. Most current studies analyze single‐cell DNA methylation data typically based on cell‐by‐region matrix [3, 4, 5]. One simple yet effective method for scDNAm data creating cell‐by‐region matrices is genome window binning, which aggregates signals and simplifies the analysis [3, 6, 7, 8]. By binning the genome into tiles of fixed lengths, such as 100 kbp, and computing the average DNA methylation level for each cell in each region, a cell‐by‐region methylation matrix can be constructed.

Before conducting downstream analyses, a critical issue must still be addressed: handling the not available (NA) values inherent in scDNAm data. For single‐cell RNA sequencing (scRNA‐seq) or single‐cell assay for transposase‐accessible chromatin using sequencing (scATAC‐seq), dropouts in sequencing lead to read counts of zeros [5]. However, in scDNAm data, captured methylation sites typically display a binary characteristic: methylated (read count of 1) or unmethylated (read count of 0), whereas uncaptured sites are NA (Figure 1A). When constructing a cell‐by‐region matrix using the window binning strategy, due to the uneven distribution of methylation sites across the genome and the effect of window size, a considerable number of regions will still have no captured methylation sites and have average methylation level marked as NA values (referred to as missing values). A methylation matrix with NA values is not permitted for downstream analysis, making the imputation of the methylation matrix a necessary preprocessing step (Figure 1A). Although there exist various imputation methods designed for other types of single‐cell sequencing data, including methods such as scCASE [9] and stPlus [10], these approaches are not applicable to the imputation of single‐cell DNA methylation data because the nature of missing data differs significantly between scDNAm data and other single‐cell omics data.

**FIGURE 1:**
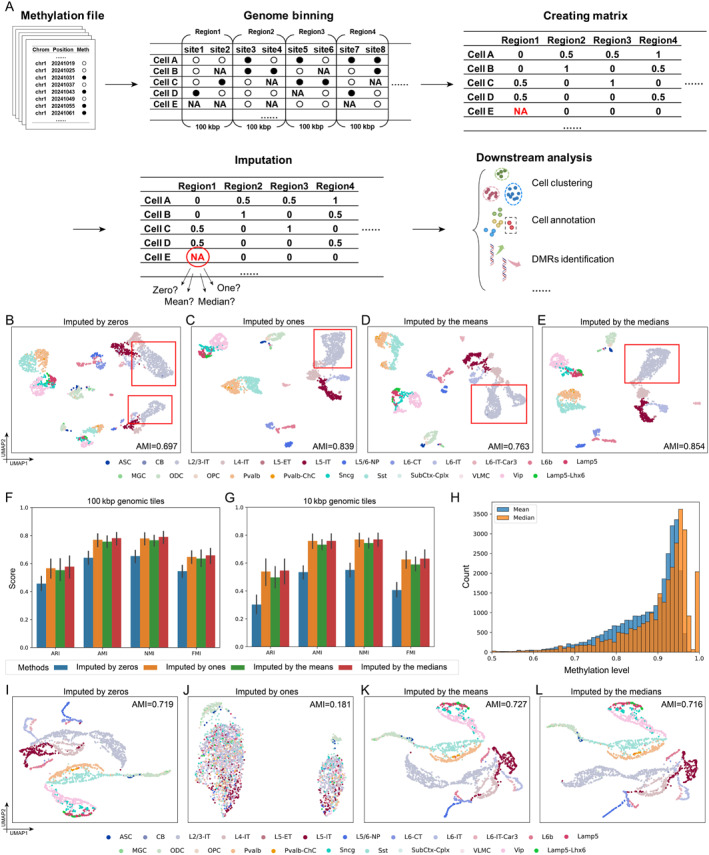
The effect of various imputation strategies. (A) Workflow of single‐cell DNA methylation data preprocessing. (B–E) UMAP visualizations of the CG methylation on “GSE167577” dataset with a region length of 100 kbp imputed by (B) zeros, (C) ones, (D) the means, and (E) the medians. (F) Clustering performance across 11 CG methylation datasets, evaluated using various metrics for different imputation strategies at tiles of 100 kbp. (G) Clustering performance across 11 CG methylation datasets, evaluated using various metrics for different imputation strategies at tiles of 10 kbp. (H) Boxplot illustrating the distribution of the means and the medians of the “GSE167577” dataset at a region length of 100 kbp. (I–L) UMAP visualizations and clustering performance of the CH methylation on “GSE167577” dataset with a region length of 100 kbp imputed by (I) zeros, (J) ones, (K) the means, and (L) the medians. UMAP, uniform manifold approximation and projection.

When analyzing scDNAm data in a manner analogous to scRNA‐seq data and scATAC‐seq data, an intuitive solution would be imputing all NA values as zeros. For example, Luo et al. [6] and Acharya et al. [11] impute regions without any DNA methylation signal as zeros in their data processing pipelines. However, from an alternative perspective, in scRNA‐seq data, higher read counts correspond to higher gene expression levels, whereas gene expression is strongly negatively correlated with DNA methylation levels [8]. The dropouts in scRNA‐seq data often indicate a high methylation level; therefore, the dropout values in scRNA‐seq data are treated as zero, which is equivalent to imputing the NA values in scDNAm data as ones. Additionally, utilizing various statistical measures to smooth NA values presents an intuitive approach. EpiScanpy imputes NA values using the means of methylation levels in a region across all cells, thus avoiding NA issues [4]. Although these studies provide several imputation strategies (Table S1), in our research, we found that imputing with the median can more effectively impute the NA values in scDNAm data and improve downstream analysis workflows.

We conducted comprehensive tests on 11 datasets with different sources, protocols, and species to evaluate the effect of various imputation strategies of scDNAm data [3, 8] (Table S2). Following the DNA methylation data analysis workflow of EpiScanpy, we created methylation matrices for each dataset using window binning of a fixed length at the whole‐genome level [4]. Subsequently, we applied four different imputation strategies to impute the NA values: using zeros, ones, means, and medians (Supporting Information S1: Supplementary Note S1). We then performed dimensionality reduction using principal component analysis (PCA) to reduce the data to 50 dimensions and applied the Louvain algorithm with default parameters for unsupervised cell clustering. An effective imputation strategy should better characterize cellular heterogeneity and improve clustering as well as other downstream analyses. Since the datasets were properly annotated, we adopted widely used metrics including the adjusted rand index (ARI), adjusted mutual information (AMI), normalized mutual information, and Fowlkes–Mallows index to evaluate clustering performance (Supporting Information S1: Supplementary Note S2).

CG methylation is the most common type of DNA methylation in vertebrates, research on CG methylation is a primary focus in current studies [1]. Thus, we first investigated the performance of different imputation strategies on CG methylation data. Following Tian et al., we first generated cell‐by‐region matrices with a region length of 100 kbp [8]. We used uniform manifold approximation and projection to visualize the embeddings obtained by PCA from the data imputed by different methods. Taking the “GSE167577” dataset as an example, the visualization of the data imputed by zeros or the means showed that many cell types, such as L4‐IT, L5‐IT, and L6‐IT, were incorrectly split into two distinct clusters (Figure 1B,C). Moreover, data imputed by the medians or ones successfully distinguished the same cell types together and accurately captured the heterogeneity among cells from different cell types (Figure 1D,E). The clustering results at 100 kbp tiles of CG methylation in 11 datasets also showed that imputation using the medians and ones yielded the best performance. Across various metrics, data imputed by the medians showed 2% advantage over the data imputed by ones (Figure 1F). Moreover, imputation by the means performed slightly worse, with the medians outperforming the means by 4.56% in ARI and 3.27% in AMI (Figure 1F). Imputation with zeros performed significantly worse than the other methods, with its clustering metrics showing a > 20% disadvantage compared to the medians (Figure 1F). Furthermore, we investigated the impact of varying cell type compositions on imputation strategies. The results demonstrate that the median‐based imputation approach consistently delivers robust and superior performance (Supporting Information S1: Supplementary Note S2, Figure S1).

To investigate imputation strategies at higher region resolutions, we reduced the region length to 10 kbp and repeated the above experiments. The results indicated that the medians and ones imputations were almost equivalent and highly effective for handling NA values at shorter tiles. However, the disadvantages of mean imputation became more pronounced at the 10 kbp level, with a 10% reduction in ARI compared to the median or one imputation (Figure 1G). Compared to the 100 kbp tiles, the 10 kbp tiles introduced more severe missing data , with most available values deviating significantly from zero (Figure 1G). As a result, using zeros to impute NA values introduced substantial erroneous signals, leading to a 44.61% drop in ARI and a 29.49% drop in AMI compared to the median imputation (Figure 1G). This indicates that the zero imputation is highly unsuitable for shorter tiles because the zero imputation in increased missing data can introduce more noise.

It is worth noting that in most single‐cell DNA CG methylation datasets, because of the high methylated proportion of CG sites, the majority of regions exhibit high methylation levels. In such cases, the median is often close to one (Figure 1H). The mean tends to be lower than the median due to the presence of differentially methylated regions (DMRs) with lower methylation levels (Figure 1H). This causes the mean to deviate from the average methylation level of NA values. Specifically, these lower methylation regions decrease the mean more significantly, whereas the median is less affected due to the relative rarity of these low‐methylation DMRs.

For CH methylation data, we used a region length of 100 kbp and conducted experiments on “GSE167577” dataset. The results of imputing with the medians for CH methylation data consistently showed favorable performance (Figure 1I–L). However, due to the extremely low methylated proportion of CH sites, using ones for imputation introduced significant noise, resulting in a complete loss of cellular heterogeneity in the imputed data (Figure 1K).

Given the above considerations and experimental results, we conclude that although imputing NA values as zeros can still reveal cell‐type specificity in scDNAm data to some extent, it is not the recommended approach for handling NA values in scDNAm data. Moreover, although imputing with means is a widely used and generally effective method, it is significantly influenced by DMRs, making it unable to accurately reflect the average methylated state of NA values. Furthermore, due to the high methylated rate of CG sites and the strong negative correlation between CG methylation level and gene expression, using ones for imputing CG methylation data is feasible. However, for CH methylation, which has an extremely low methylated rate, imputing with ones would introduce intolerable noise. In conclusion, imputing NA values with the medians best exhibits cellular heterogeneity and preserves the biological signal.

In summary, based on our findings, we suggest that imputing NA values with the median is a straightforward and effective way to highlight cellular heterogeneity in scDNAm data, offering an accurate data foundation for downstream analyses and allowing for a more accurate and reliable interpretation of the underlying biology.

## AUTHOR CONTRIBUTIONS

**Songming Tang**: Conceptualization; methodology; writing—original draft; writing—review and editing. **Siyu Li**: Data curation; writing—review and editing. **Shengquan Chen**: Conceptualization; funding acquisition; validation; writing—review and editing.

## CONFLICT OF INTEREST STATEMENT

The authors declare no conflicts of interest.

## Supporting information

Supporting Information S1

## Data Availability

All datasets in this study are publicly available under the following National Center for Biotechnology Information (NCBI) Gene Expression Omnibus (GEO) accessions: GSE130553, GSE131354, GSE131360, GSE131393, GSE131406, GSE167577, GSE168066, GSE168645, GSE168734, GSE179610, and GSE179971.
