# Supplementary material for: Imputing not available values in single‐cell DNA methylation data using the median is straightforward and effective
Source: Quant Biol. 2025 Apr 1;13(3):e70000. doi: 10.1002/qub2.70000 (PMC12806076; doi:10.1002/qub2.70000)
Supplement: Supplementary file 1 — Supporting Information S1 [file QUB2-13-e70000-s001.docx]

**Supplementary Information for**

**Imputing not available values in single-cell DNA methylation data using the median is straightforward and effective**

Songming Tang^1^, Siyu Li^1^, Shengquan Chen^1, *^

^1^ School of Mathematical Sciences and LPMC, Nankai University, Tianjin, China.

^*^ Correspondence

Shengquan Chen

Email: chenshengquan@nankai.edu.cn

**Supplementary Note S1: Details of the implementation of experiments.**

Firstly, we utilized the EpiScanpy (Version 0.4.0) package to bin the genomic sequence into multiple regions using a tile length of 100 kbp (or 10 kbp). We then calculated the average DNA methylation level within each region for individual cells to construct a cell-by-region matrix. Regions devoid of any captured sites were annotated with NA values. Secondly, we employed the NumPy (Version 1.26.4) package to remove regions where the read count was NA across 95% of all cells. Additionally, we excluded cells with undefined cell types to ensure an accurate evaluation of clustering efficacy. Finally, we addressed the NA values within the matrix by imputing them with zeros, ones, the means methylation level observed across all cells, and the median methylation level observed across all cells for a given region. We used EpiScanpy (Version 0.4.0) to calculate the means and NumPy (Version 1.26.4) to impute with zeros, ones and the medians. The specific implementation and reproducible code can be found on Zenodo^1^.

**Supplementary Note S2: Details of the evaluation metrics.**

For ARI, suppose that $C$ are the actual labels of the clusters and $K$ are the clustering labels. In that case, we define $a$ and $b$ as follows: $a$, the number of pairs of elements in the same set in $C$ and the same set in $K$. $b$, the number of pairs of elements in different sets in $C$ and different sets in $K$. The following formula then gives the original Rand index:

$$\begin{aligned} \begin{matrix} RI=\frac{a+b}{C_{2}^{n_{\text{samples }}}} \end{matrix} \#\left( 1 \right) \end{aligned}$$

$C_{2}^{n_{\text{samples }}}$is the total number of possible pairs in the datasets, and the following formula gives the ARI, which ensures random matching will get a value close to 0 and perfect matching gets a value close to 1:

$$\begin{aligned} \begin{matrix} ARI=\frac{RI-E\left[ RI \right]}{\max\left( RI \right)-E\left[ RI \right]} \end{matrix} \#\left( 2 \right) \end{aligned}$$

Mutual Information (MI) evaluates the degree of dependence between two random variables. AMI adjusts MI by considering the expected value under random clustering, as shown in Equation $\left( 3 \right)$.

$$\begin{aligned} \begin{matrix} AMI=\frac{MI\left( \mathbf{P},\mathbf{T} \right)-E\left[ MI\left( \mathbf{P},\mathbf{T} \right) \right]}{avg\left[ H\left( \mathbf{P} \right),H\left( \mathbf{T} \right) \right]-E\left[ MI\left( \mathbf{P},\mathbf{T} \right) \right]} \end{matrix} \#\left( 3 \right) \end{aligned}$$

Besides, the Fowlkes-Mallows Index (FMI) can be used as the geometric mean of pairwise precision and recall.

$$\begin{aligned} FMI=\frac{TP}{\sqrt{\left( TP+FP \right)\left( TP+FN \right)}}\#\left( 4 \right) \end{aligned}$$

**Supplementary Note S3: Clustering performance on down-sampling datasets with varying cell type compositions.**

To investigate the impact of different cell types on single-cell DNA methylation imputation strategies, we conducted multiple random samplings of cells from various cell types to assess the generalizability of the strategies across datasets with diverse cell type compositions. Specifically, we utilized the GSE167577 dataset, which comprises 23 major cell types, as an example. We randomly selected 10 cell types and applied different imputation strategies to the data, subsequently evaluating the clustering performance. We performed 25 iterations of this experiment, and the median imputation strategy consistently demonstrated superior results (Supplementary Fig. S1). Our results indicate that employing the median for imputation remains the most effective approach for facilitating cell clustering in datasets characterized by varying cell type compositions. We have incorporated the discussion of this section into Supplementary Note S3 and Supplementary Fig. S1.


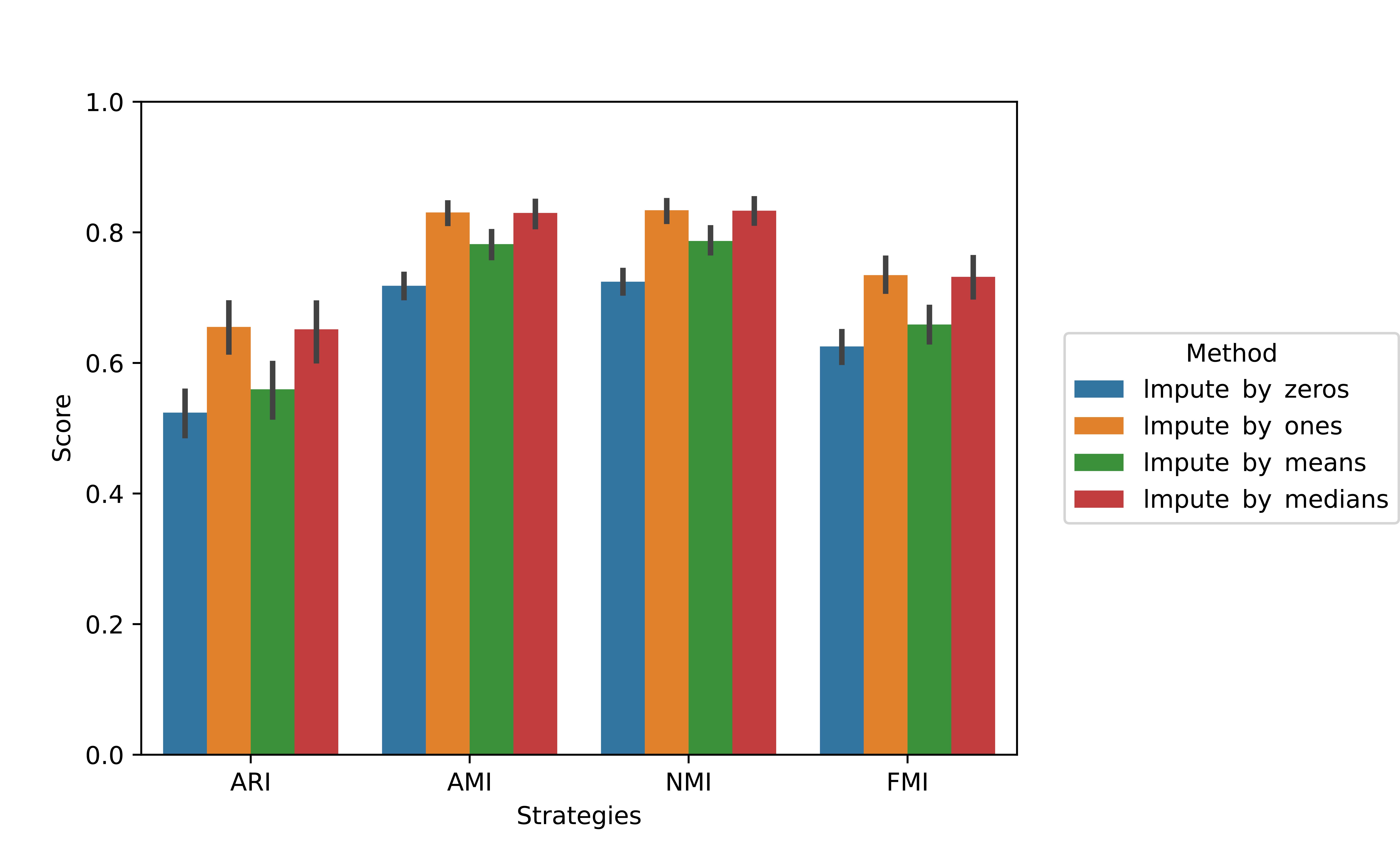


**Supplementary Figure S1.** **Clustering performance on the GSE167577 dataset, evaluated on down-sampling subsets containing ten randomly selected cell types, with the process repeated 25 times for robust assessment.**

**Supplementary Table S1.** Summary of the baseline imputation strategies.

| **Strategy** | **Sequencing method** | **Length of tiles** | **Year** | **Reference** |
| --- | --- | --- | --- | --- |
| Zeros | snmC-seq2 | 100 kbp | 2018 | Luo, C. et al.^2^ |
|  | sciMETv2 | 50 kbp | 2024 | Acharya, Sonia N. et al.^3^ |
| Ones | snmC-seq3 | 100 kbp | 2023 | Tian, W. et al.^4^ |
| Means | NA | 100 kbp | 2021 | Danese, A. et al.^5^ |

**Supplementary Table S2.** Summary of the 11 CG methylation datasets used in this study.

| **Dataset** | **Species** | **Sequencing method** | **No. of cells** | **No. of cell types** | **NA values**  **proportion (100 kbp)** | **NA values**  **proportion (10 kbp)** | **Literature** |
| --- | --- | --- | --- | --- | --- | --- | --- |
| GSE130553 | *Mus musculus* | snmC-seq2 | 1367 | 22 | 2.94% | 19.47% | Liu, H. et al.^6^ |
| GSE131354 | *Mus musculus* | snmC-seq2 | 296 | 21 | 4.55% | 16.33% | Liu, H. et al.^6^ |
| GSE131360 | *Mus musculus* | snmC-seq2 | 1262 | 22 | 5.73% | 18.88% | Liu, H. et al.^6^ |
| GSE131393 | *Mus musculus* | snmC-seq2 | 1106 | 26 | 2.85% | 21.96% | Liu, H. et al.^6^ |
| GSE131406 | *Mus musculus* | snmC-seq2 | 1433 | 24 | 2.84% | 26.50% | Liu, H. et al.^6^ |
| GSE167577 | *Homo sapiens* | snmC-seq3 | 2518 | 23 | 2.24% | 33.37% | Tian, W. et al.^4^ |
| GSE168066 | *Homo sapiens* | snmC-seq3 | 2496 | 21 | 2.23% | 31.83% | Tian, W. et al.^4^ |
| GSE168645 | *Homo sapiens* | snmC-seq3 | 2951 | 22 | 2.39% | 35.96% | Tian, W. et al.^4^ |
| GSE168734 | *Homo sapiens* | snmC-seq3 | 2496 | 21 | 2.23% | 30.64% | Tian, W. et al.^4^ |
| GSE179610 | *Homo sapiens* | snmC-seq3 | 2786 | 20 | 1.69% | 22.53% | Tian, W. et al.^4^ |
| GSE179971 | *Homo sapiens* | snmC-seq3 | 2730 | 25 | 2.29% | 34.89% | Tian, W. et al.^4^ |

References

1. Tang, S., Li, S. & Chen, S. Imputing not available values in single-cell DNA methylation data using the median is straightforward and effective. *Zenodo* https://doi.org/10.5281/zenodo.14601044 (2025).

2. Luo, C. et al. Robust single-cell DNA methylome profiling with snmC-seq2. *Nature Communications* **9**, 3824 (2018).

3. Acharya, S.N. et al. sciMET-cap: High-throughput single-cell methylation analysis with a reduced sequencing burden. *Genome Biology* **25**, 186 (2024).

4. Tian, W. et al. Single-cell DNA methylation and 3D genome architecture in the human brain. *Science* **382**, eadf5357 (2023).

5. Danese, A. et al. EpiScanpy: integrated single-cell epigenomic analysis. *Nature Communications* **12**, 5228 (2021).

6. Liu, H. et al. DNA methylation atlas of the mouse brain at single-cell resolution. *Nature* **598**, 120-128 (2021).
